# Combined Use of Tocilizumab and Mesenchymal Stem Cells Attenuate the Development of an Anti-HLA-A2.1 Antibody in a Highly Sensitized Mouse Model

**DOI:** 10.3390/ijms25031378

**Published:** 2024-01-23

**Authors:** Xianying Fang, Sheng Cui, Hanbi Lee, Ji Won Min, Sun Woo Lim, Eun-Jee Oh, Chul Woo Yang, Yoo Jin Shin, Byung Ha Chung

**Affiliations:** 1 Transplantation Research Center, College of Medicine, The Catholic University of Korea, Seoul 06591, Republic of Korea; xianyingfang@catholic.ac.kr (X.F.); cuishengmd@catholic.ac.kr (S.C.); hanbilee89@catholic.ac.kr (H.L.); blueberi12@gmail.com (J.W.M.); swlim@catholic.ac.kr (S.W.L.); ejoh@catholic.ac.kr (E.-J.O.); yangch@catholic.ac.kr (C.W.Y.); 2 Division of Nephrology, Department of Internal Medicine, Seoul St. Mary’s Hospital, The College of Medicine, The Catholic University of Korea, Seoul 06591, Republic of Korea; 3 Division of Nephrology, Department of Internal Medicine, Bucheon St. Mary’s Hospital, The College of Medicine, The Catholic University of Korea, Bucheon-si 14647, Republic of Korea; 4 Department of Laboratory Medicine, Seoul St. Mary’s Hospital, The College of Medicine, The Catholic University of Korea, Seoul 06591, Republic of Korea

**Keywords:** HLA.A2 transgenic mice, sensitization, skin allograft, tocilizumab, mesenchymal stem cells

## Abstract

Sensitization to HLA can result in allograft loss for kidney transplantation (KT) patients. Therefore, it is required to develop an appropriate desensitization (DSZ) technique to remove HLA-donor-specific anti-HLA antibody (DSA) before KT. The aim of this research was to investigate whether combined use of the IL-6 receptor-blocking antibody, tocilizumab (TCZ), and bone-marrow-derived mesenchymal stem cells (BM-MSCs) could attenuate humoral immune responses in an allo-sensitized mouse model developed using HLA.A2 transgenic mice. Wild-type C57BL/6 mice were sensitized with skin allografts from C57BL/6-Tg (HLA-A2.1)1Enge/J mice and treated with TCZ, BM-MSC, or both TCZ and BM-MSC. We compared HLA.A2-specific IgG levels and subsets of T cells and B cells using flow cytometry among groups. HLA.A2-specific IgG level was decreased in all treated groups in comparison with that in the allo-sensitized control (Allo-CONT) group. Its decrease was the most significant in the TCZ + BM-MSC group. Regarding the B cell subset, combined use of TCZ and BM-MSC increased proportions of pre-pro B cells but decreased proportions of mature B cells in BM (*p* < 0.05 vs. control). In the spleen, an increase in transitional memory was observed with a significant decrease in marginal, follicular, and long-lived plasma B cells (*p* < 0.05 vs. control) in the TCZ + BM-MSC group. In T cell subsets, Th2 and Th17 cells were significantly decreased, but Treg cells were significantly increased in the TCZ+BM-MSC group compared to those in the Allo-CONT group in the spleen. Regarding RNA levels, IL-10 and Foxp3 showed increased expression, whereas IL-23 and IFN-γ showed decreased expression in the TCZ + BM-MSC group. In conclusion, combined use of TCZ and BM-MSC can inhibit B cell maturation and up-regulate Treg cells, finally resulting in the reduction of HLA.A2-specific IgG in a highly sensitized mouse model. This study suggests that the combined use of TCZ and BM-MSC can be proposed as a novel strategy in a desensitization protocol for highly sensitized patients.

## 1. Introduction

Kidney transplantation (KT) is the optimal therapeutic option for patients with end-stage kidney disease [1,2,3,4]. However, the presence of an allo-antibody to human leukocyte antigen (HLA), the so-called “sensitization to HLA”, is still a significant obstacle to successful KT [2]. KT in patients with strong donor-specific anti-HLA antibodies (HLA-DSAs) without any treatment can induce severe antibody-mediated rejection (ABMR), which can result in immediate allograft loss. The presence of weak HLA-DSA at baseline is even associated with a higher risk for the development of ABMR and lower allograft survival [5,6,7,8]. Therefore, proper “desensitization (DSZ) therapy” to remove HLA-DSA before KT is very crucial [9,10]. 

The currently used desensitization technique is performed by removing preexisting antibodies and inhibiting the production of allo-antibodies. Several desensitization protocols, such as the use of plasmapheresis, rituximab, intravenous immune globulin (IVIg), and bortezomib, are being applied to these patients [9,10]. Recently, it has been reported that IL-6 targeting therapy shows promising results in suppressing anti-HLA allo-antibody in both animal studies and kidney transplant recipients [11,12]. For example, a treatment protocol including tocilizumab (TCZ), an IL-6 receptor-blocking antibody, is effective for DSZ or for treating chronic antibody-mediated rejection [13,14,15,16,17]. Especially for DSZ, TCZ is not used as a monotherapy. It is combined with other therapeutics in most studies because using TCZ alone has limited effectiveness for DSZ. 

Meanwhile, mesenchymal stem cells (MSCs) have been proposed as a new therapy to suppress allo-immune responses in KT using their immunomodulatory properties [18,19,20]. Indeed, many previous studies have reported that MSCs can significantly affect many types of immune cells constituting the immune system through multiple mechanisms, such as the secretion of regulatory cytokines and the activation of regulatory immune cells [21,22]. Some animal and clinical studies have also shown promising results for MSCs as novel therapeutics for suppressing allo-immune responses in KT [19,23]. However, the efficacy of MSCs is still compromised by their limited colonization rate and short survival time within injured organs. Therefore, in most previous studies using MSCs in KT, other immune suppressive agents were concomitantly applied with MSCs [23,24].

Based on the above background, the objective of this study is to investigate the efficacy of combined or individual use of TCZ or MSCs in preventing the production of anti-HLA-A2 allo-antibody using a well-established sensitized mouse model [1]. 

## 2. Results

### 2.1. Comparison of Anti-HLA.A2 Antibody Responses in Sensitized Mice after Treatment 

**Figure 1:**
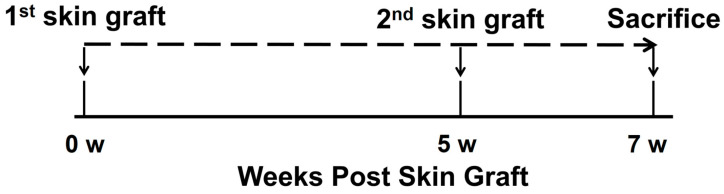
Study protocol. We performed skin grafts from HLA-A2 mice into normal B6 mice. These mice were homozygous. They carried the Tg HLA-A2.1 Enge transgene, thereby expressing human class I MHC-Ag HLA-A2.1. Serum samples were collected at weeks 0 and 7 from skin graft recipients. A primary skin graft was performed for all groups. The second skin graft was performed at week 5. Mice were sacrificed at week 7. Spleen and bone marrow from femoral bone were harvested.

**Figure 2:**
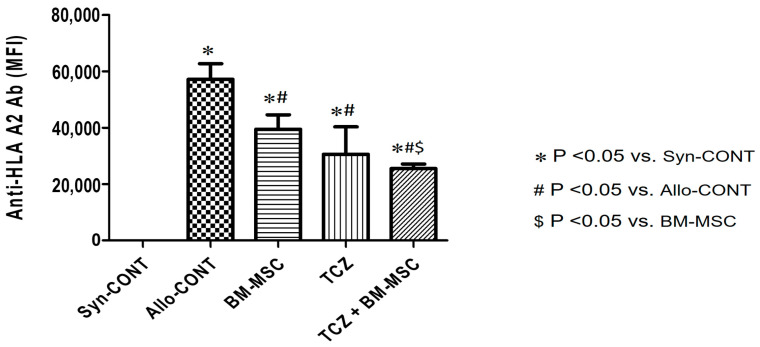
Comparison of the level of HLA.A2-specific IgG. MFI titers of serum donor-reactive HLA.A2-specific IgG were measured at weeks 0, 5, and 7. Ab was hardly detected in the syngeneic CONT group, whereas its titers reached 30–40 thousand by week 7 in the other three groups (BM-MSC, TCZ, and BM-MSC+TCZ). Abbreviations: MFI, median fluorescence intensity.

To investigate whether combined use of TCZ and BM-MSC could decrease the generation of anti-HLA-A2 allo-antibody, we used a sensitized mouse model (Figure 1) [1]. DSA responses in skin graft recipient plasma samples were observed at week 0 (before the first skin graft) and week 7 (7 weeks after the first skin graft). The anti-human HLA-A2.1 antibody (MHC class I) was detected and presented as a median fluorescence intensity (MFI) titer (Figure 2). Results showed significantly increased levels of MFI titers in the Allo-CONT group (57,384 ± 8715) compared with that of the Syn-CONT group (26.28 ± 0.60). However, MFI titers decreased in all three treated groups compared to the Allo-CONT group (BM-MSC group, 40,334 ± 6343; TCZ group, 34,604 ± 18,032; and TCZ + BM-MSC group, 25,155 ± 3738). The MFI titer was the lowest in the TCZ + BM-MSC group (Figure 2).

### 2.2. Comparison of B Cell Fractions in Bone Marrow 

**Figure 3:**
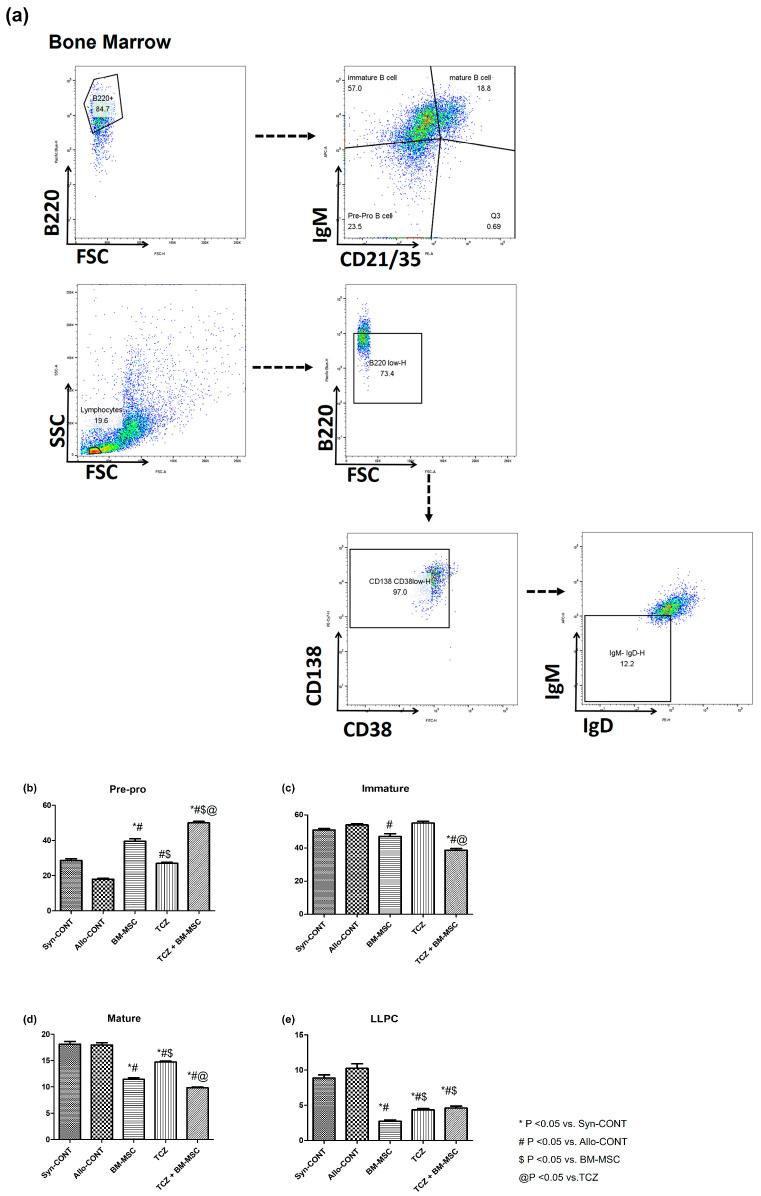
B cell subset in the BM. The B cell population at week 7 (2 weeks after second transplantation) in the recipient bone marrow was analyzed using flow cytometry. (**a**) Gating strategy; (**b**) proportions of B220+CD21/CD35-IgM-pre-pro B cells; (**c**) proportions of B220+CD21/CD35-IgM+ immature B cells; (**d**) proportions of B220+CD21/CD35+IgM+ mature B cells; and (**e**) proportions of B220lowCD138+CD38low (Ig–) long-lived plasma cells (LLPC). N = 3 animals/group.

To examine the effects of treatment with BM-MSC alone, TCZ alone, or their combination on B cell subsets, we analyzed the proportion of B cells using flow cytometry (Figure 3a). Pre-pro B cells were significantly increased in treated groups compared to those in the Allo-CONT group. They showed the most increase in the TCZ + BM-MSC group (Syn-CONT: 28.64 ± 0.977%; Allo-CONT: 17.97 ± 0.433%; BM-MSC: 39.60 ± 1.342%; TCZ: 27.00 ± 0.622%; TCZ + BM-MSC: 49.95 ± 0.939%) (Figure 3b). In contrast, proportions of immature and mature B cells decreased in all treated groups compared to those in the Allo-CONT group. They showed the most reduction in the TCZ + BM-MSC group (Immature B cells: Syn-CONT, 50.94 ± 0.884%; Allo-CONT, 53.99 ± 0.7609%; BM-MSC, 47.05 ± 1.606%; TCZ, 55.13 ± 1.088%; TCZ + BM-MSC, 38.80 ± 0.958%; Mature B cells: Syn-CONT, 18.13 ± 0.51%; Allo-CONT, 17.97 ± 0.43%; BM-MSC, 11.46 ± 0.28%; TCZ, 14.74 ± 0.19%; TCZ + BM-MSC, 9.830 ± 0.18%) (Figure 3c,d). In addition, long-lived plasma cells (LLPCs) were significantly decreased in all treated groups compared to those in the Allo-CONT group (Syn-CONT, 8.870 ± 0.47%; Allo-CONT, 10.26 ± 0.62%; BM-MSC, 2.748 ± 0.19%; TCZ, 4.342 ± 0.21%; TCZ + BM-MSC, 4.608 ± 0.28%) (Figure 3e).

### 2.3. Comparison of B Cell Fractions in the Spleen 

**Figure 4:**
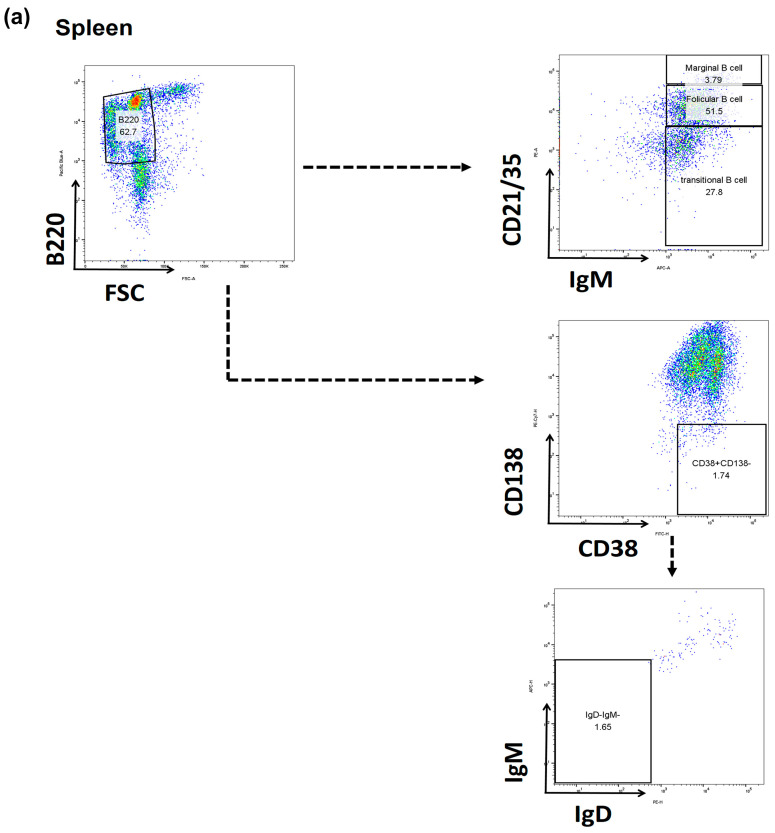

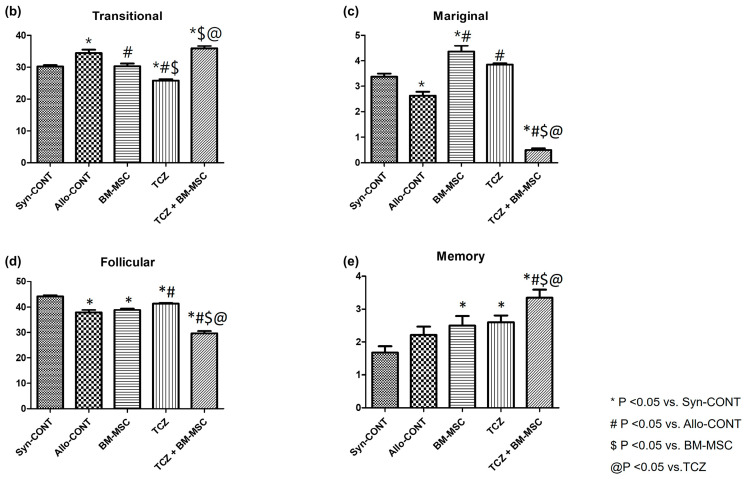
B cell subset in the spleen. The B cell population at week 7 (2 weeks after second transplantation) in the spleen was analyzed using flow cytometry. (**a**) Gating strategy; (**b**) proportions of B220+CD21/CD35loIgM+ transitional B cells; (**c**) proportions of B220+CD21/CD35+IgM+ marginal B cells; (**d**) proportions of B220+CD21/CD35hiIgM+ follicular B cells; and (**e**) proportions of B220+CD138-CD38+IgM-IgD-memory B cells. N = 3 animals/group.

Furthermore, we observed proportions of B cells in spleens using flow cytometry (Figure 4a). Transitional B cells were decreased in the BM-MSC and TCZ groups compared to those in the Allo-CONT group. However, they were increased in the TCZ + BM-MSC group (Syn-CONT, 30.26 ± 0.43%; Allo-CONT, 34.42 ± 1.03%; BM-MSC, 30.35 ± 0.79%; TCZ, 25.77 ± 0.44%; TCZ + BM-MSC, 35.89 ± 0.75%) (Figure 4b). On the other hand, marginal B cells were increased in BM-MSC and TCZ groups compared to those in the Allo-CONT group, with the most reduction being observed in the TCZ + BM-MSC group (Syn-CONT, 3.372 ± 0.13%; Allo-CONT, 2.636 ± 0.15%; BM-MSC, 4.364 ± 0.23%; TCZ, 3.857 ± 0.046%; TCZ + BM-MSC, 0.50 ± 0.06%) (Figure 4c). Proportions of follicular B cells were significantly decreased, whereas those of memory B cells were increased in the TCZ + BM-MSC group compared to those in other groups (Follicular B cells: Syn-CONT, 44.15 ± 0.45%; Allo-CONT, 37.83 ± 1.01%; BM-MSC, 38.85 ± 0.54%; TCZ, 41.30 ± 0.26%; TCZ + BM-MSC, 29.69 ± 0.83%; Memory B cells: Syn-CONT, 1.679 ± 0.19%; Allo-CONT, 2.218 ± 0.25%; BM-MSC, 2.498 ± 0.29%; TCZ, 2.603 ± 0.20%; TCZ + BM-MSC, 3.344 ± 0.25%) (Figure 4d,e).

### 2.4. Comparison of T Cell Fractions in the Spleen 

**Figure 5:**
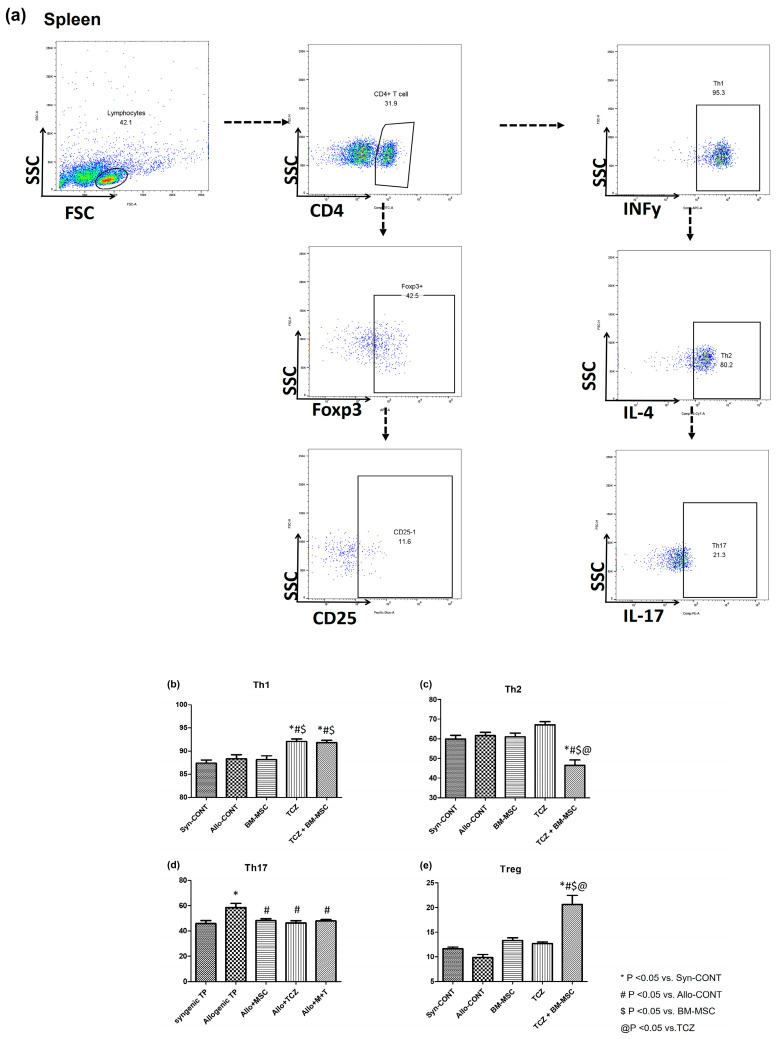
T cell subset in the spleen. The B cell population at week 7 (2 weeks after second transplantation) in the spleen was analyzed using flow cytometry. (**a**) Gating strategy; (**b**) proportions of CD4+/INFγ+ Th1 cells; (**c**) proportions of CD4+/IL4+ Th2 cells; (**d**) proportions of CD4+/IL-17+ Th17 cells; and (**e**) proportions of CD4+/CD25+/Foxp3+ Treg cells. N = 3 animals/group.

To investigate the effects of BM-MSC alone, TCZ alone, and their combination on T cell activation and B cell activation, we analyzed T cells in the spleen using flow cytometry (Figure 5a). Results showed that proportions of Th l cells were increased in TCZ and TCZ + BM-MSC groups compared to those in other groups (Syn-CONT, 87.41 ± 0.67%; Allo-CONT, 88.35 ± 0.89%; BM-MSC, 88.15 ± 0.84%; TCZ, 92.09 ± 0.55%; TCZ + BM-MSC, 91.81 ± 0.55%) (Figure 5b). Proportions of Th2 cells showed the greatest decrease in the TCZ + BM-MSC group compared to other groups (Syn-CONT, 59.90 ± 1.18%; Allo-CONT, 61.61 ± 1.704%; BM-MSC, 60.98 ± 1.98%; TCZ, 67.14 ± 1.60%; TCZ + BM-MSC, 43.19 ± 3.038%) (Figure 5c). Proportions of Th17 cells were significantly decreased in drug-treated groups compared to those in Allo-CONT groups, whereas those of Treg cells were significantly increased in drug-treated groups, with the TCZ + BM-MSC group showing the most significant increase of Treg cells (Th17: Syn-CONT, 45.93 ± 2.242%; Allo-CONT, 58.51 ± 3.29%; BM-MSC, 48.18 ± 1.41%; TCZ, 46.37 ± 1.737%; TCZ + BM-MSC, 47.91 ± 1.00%; Treg: Syn-CONT, 11.62 ± 0.38%; Allo-CONT, 9.85 ± 0.60%; BM-MSC, 13.29 ± 0.57%; TCZ, 12.68 ± 0.31%; TCZ + BM-MSC, 20.60 ± 1.87%) (Figure 5d,e).

### 2.5. mRNA Expression of Cytokines after Treatment with BM-MSC and TCZ in a Highly Sensitized Mouse Model

**Figure 6:**
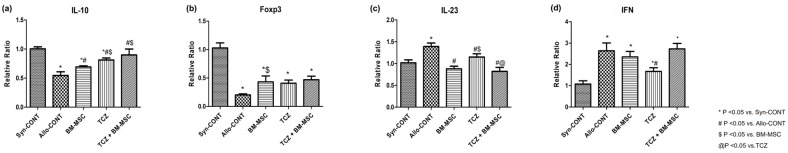
Quantitative real-time PCR. The mRNA expression of cytokines was detected with RT-qPCR in the spleen of sensitized mice. Relative mRNA expression levels were calculated after normalization to β-actin expression. Expression level in the Syn-CONT group was considered a control. Values shown are presented as mean ± SE (*n* = 3).

RT-qPCR analysis revealed that mRNA levels of IL-10 and Foxp3 were significantly increased in drug-treated groups compared with those in Allo-CONT groups (Figure 6a,b). In contrast, IL-23 and IFN mRNA levels were decreased in drug-treated groups compared with those in Allo-CONT groups (Figure 6c,d). IFN showed lower expression in the TCZ group than in the Allo-CONT group, although it showed no significant difference between the TCZ + BM-MSC group and the Allo-CONT group.

## 3. Discussion

In this study, we found that BM-MSC and TCZ showed significant suppressive effects on the development of anti-HLA-A2 Ab, with the combined use of BM-MSC and TCZ showing the most significant efficacy in a well-established highly sensitized mouse model [1,2]. In addition, suppression of anti-HLA-A2 antibody formation was accompanied by up-regulation of regulatory T cells in the spleen and expression levels of associated cytokines. Our results suggest that the combined use of BM-MSC and TCZ can be proposed as a potential therapeutic strategy for desensitization in highly sensitized patients waiting for transplantation. 

First, we compared anti-HLA-A2 Ab titers according to the use of BM-MSC with or without TCZ in a highly sensitized mouse model. We performed two skin grafts to establish the sensitization model. In theory, memory B cells for HLA.A2 are formed upon first exposure to a specific antigen. To achieve a “highly sensitized state”, we proceed with exposure to a second antigen to induce the expansion and differentiation of these memory B cells into antibody-secreting plasma cells [2,9]. We compared anti-HLA-A2 Ab titers using samples taken when we sacrificed mice 2 weeks after the second skin graft. As expected, anti-HLA.A2 Ab was not detected in the non-sensitized Syn-CONT group. In allogeneic transplant groups (Allo-CONT), a high titer of anti-HLA.A2 Ab was detected, similar to the findings of previous studies [1,9]. In contrast, antibody levels were significantly decreased in all treated groups (BM-MSC, TCZ, and TCZ + BM-MSC), suggesting a suppressive effect of BM-MSC or TCZ on anti-HLA.A2 Ab formation. 

Second, to identify the cellular mechanisms for the suppression of anti-HLA.A2 Ab formation, we examined the immune cells, which consist of B cell and T cell lineages, in the spleen and bone marrow using flow cytometry. The most significant results of the immune cell analysis were the decrease of Th17 cells and the increase of Treg cells in the TCZ + BM-MSC groups. The proportion of Th17 cells in the spleen showed an increase in the ALLO-CONT group in comparison with the SYN-CONT group but showed a decrease in all three treated groups. In contrast, the proportion of Treg cells showed a decreasing pattern in the ALLO-CONT group in comparison with the SYN-CONT group but recovered in the BM-MSC or TCZ-treated group. Of note, the increase of Treg cells was the most significant in the TCZ + BM-MSC group. We also found that the expression of IL-23 mRNA, a signature cytokine for the Th17 pathway, was decreased, whereas IL-10 and FOXP3 mRNAs as regulatory immune cell markers were increased in the TCZ + BM-MSC group. 

Several studies, including ours, have reported that Th17 cells and their signature cytokines might play an important role in the activation of the humoral immune system and the progression of allograft injury [5,6]. Furthermore, an increase in the number of Treg cells is an important factor in achieving immune tolerance, while a decrease in Treg cells is closely associated with antibody-mediated rejection [10,11]. Increased expression of IL-17 on tubular epithelial cells has been found in kidney transplant recipients with acute antibody-mediated rejection [12]. Our previous studies have also found that an increased infiltration of Th17 in allograft tissues or an increase in the proportion of Th17 cells in peripheral blood compared to Treg cells is associated with more severe allograft rejection or allograft dysfunction [13,14,18]. Accumulating evidence has found that balancing Th17/Treg is an effective strategy for preventing the formation of allo-antibody or for treating antibody-mediated allograft injury [18,19].

Both TCZ and MSCs have shown beneficial effects in terms of up-regulating Treg cells and inhibiting Th17 cells in many previous studies. Neutralization of IL-6 can reduce allograft rejection by allowing the emergence of Tregs and inhibiting Th17 cells [21,22]. It has been shown that increased Treg differentiation is related to significant reductions of anti-IL-6R antibodies in graft-to-host disease and allograft rejection in animal models [23,24]. In addition, the high-affinity TopoI reactive B cells can induce Th17 cells by producing IL-6 and IL-23 in an autoimmune disease murine model. It has also been shown that low-affinity TopoI reactive B cells induce Tregs via inhibitory cytokines such as IL-10 and IL-35 [25]. Furthermore, MSCs can remodel the balance between effector and regulatory immune cells, result in a decrease in the release of interleukin-17 (IL-17) by Th17 cells, and facilitate the emergence of Treg cells [26,27,28]. Of note, previous studies have shown that the combination of an IL-6 receptor antagonist and MSCs can synergistically lead to an increase in Tregs [29,30]. Therefore, it is possible that the combined use of TCZ and BM-MSCs can synergistically increase the proportion of Tregs and decrease Th17 cells in this study. 

With changes in T cell subsets, B cell subsets in BM and spleen also showed significant changes after treatment with TCZ in the presence or absence of BM-MSCs treatment. In BM B cell subset analysis, mature B cells and LLPC, which could be associated with the activated B cells, and production levels of allo-antibody were deceased in all three drug-treated groups in comparison with ALLO-CONT groups [31]. In contrast, pre-pro B cells increased in drug-treated groups, especially in the BM-MSC group or the TCZ + BM-MSC group. The reason for this finding was unclear. Pre-pro B cells might have increased as compensation for the decrease of LLPC or mature B cells in drug-treated groups. Regarding spleen B cells, marginal B cells or follicular B cells decreased the most in the TCZ + BM-MSC group. Both cells are known to play a role in activating humoral immune responses in the secondary lymphoid organ with the help of T cells [32]. Thus, decreases in these cell types might be associated with a decreased production of anti-HLA.A2 antibodies in the TCZ + BM-MSC group. 

This study has some limitations. First, a major limitation of our study was the small number of animals per group. We performed repeated experiments for internal validation and quality control. Another limitation was that even though we observed changes in T cell and B cell subsets that could be associated with a decrease in allo-antibody production, we could not clearly reveal relationships between changes in immune cells by TCZ and BM-BMC treatment. Sophisticated high-throughput research, such as single-cell RNA studies, should be conducted in the future to clarify such relationships. 

In conclusion, we found that the combined use of BM-MSC and TCZ could suppress the development of anti-HLA-A2 antibodies. The underlying mechanism was associated with the upregulation of Treg. This study suggests that BM-MSC, in addition to TCZ, can be proposed as a new strategy in a desensitization protocol for highly sensitized patients. 

## 4. Materials and Methods

### 4.1. Animals

In this study, 8- to 12-week-old homozygous C57BL/6-Tg (HLA-A2.1)1Enge/J mice (Jackson Laboratory, Bar Harbor, ME, USA; jaxmice.jax.org) and wild-type male C57BL/6 mice (Orient Bio, Seongnam, Republic of Korea) initially weighing 25–30 g were used. All mice were housed in cages (Nalge, Rochester, NY, USA) (five animals/cage) under controlled conditions with a temperature of 20–26 °C, a humidity of 50 ± 10%, and a 12 h/12 h light/dark cycle at the animal care facility of the Catholic University of Korea. All animal procedures and experiments were approved by the Institutional Animal Care and Use Committee (IACUC) of the School of Medicine, Catholic University of Korea (CUMC-2022-0115-02). They were conducted in accordance with the Laboratory Animal Welfare Act, the Guide for the Care and Use of Laboratory Animals, and Guidelines and Policies for Rodent Experiments.

### 4.2. Skin Allograft Transplant Procedure

For the experimental sensitization model, a previously described murine skin graft protocol [11] was employed. Briefly, C57BL/6-Tg (HLA-A2.1)1Enge/J mice were used as donors for skin transplantation. Skin from the donor tail (1.0 cm^2^) was grafted onto the backs of wild-type C57BL/6 recipients. Except for a single transgenic HLA.A2 antigen, both donor and recipient strains had a common genetic B6 background. HLA.A2 antigen expression in donor cells induced an alloimmune response after transplantation and led to the production of HLA.A2-specific antibodies (anti-HLA.A2 antibodies) in recipients.

### 4.3. Preparation of Human Bone Marrow-Derived Mesenchymal Stem Cells 

We used human bone marrow-derived MSCs (BM-MSC) (Catholic MASTER Cells) from the Catholic Institute of Cell Therapy (CIC, Seoul, Republic of Korea). These Catholic MASTER cells were certified as allogeneic MSCs by the KFDA. These cells were obtained by the aspiration of the human bone marrow from the iliac crests of healthy donors aged between 20 and 55 years. The procedures were approved by the Institutional Review Board of Seoul St. Mary’s Hospital (approval numbers: KIRB-00344-009 and KIRB-00362-006). Collected bone marrows were sent to the CIC under good manufacturing practice (GMP) conditions. The CIC was responsible for the isolation, expansion, and quality control of the allogeneic MSCs. The characterization of Catholic MASTER cells has been reported in a previous study [33]. Briefly, cells were expanded and tested for multilineage differentiation and cell-surface antigens in conditions of bacterial sterility, mycoplasma sterility, and a low endotoxin level (<3 EU/mL) in a GMP-compliant facility.

### 4.4. Experimental Design

After 1-week acclimation, weight-matched mice were randomized into the following five groups (n = 3 per group), as shown in Figure 1: (1) a syngeneic skin graft control (Syn-CONT) group that received syngeneic transplants (from B6 to B6 mice); (2) an allogeneic skin graft control (Allo-CONT) group that received allogeneic transplants twice (from C57BL/6-Tg (HLA-A2.1)1Enge/J to B6 mice); (3) a BM-MSC group that received allogeneic skin graft transplants and treatment with 2 × 10^5^ of BM-MSC/mouse by tail vein injection once a week; (4) a tocilizumab (TCZ) group that received allogeneic skin graft transplants and treatment with 10 mg/kg of TCZ (ACTEMRA tocilizumab, JW pharmaceutical, Gwacheon, Republic of Korea)/mouse by intraperitoneal injection three times a week; and (5) a TCZ + BM-MSC group that received allogeneic skin graft transplants and treatment with BM-MSC and TCZ (Table S1). Mice were sacrificed 7 weeks after transplantation. Spleen cells and bone marrow from femoral bone were collected (Figure 1).

### 4.5. Measurement of Serum Donor-Specific Anti-HLA.A2 Antibodies 

Blood samples were collected from facial veins in all five groups before the 1st skin graft implantation and at sacrifice (7 weeks from the 1st graft). The analysis of donor-specific anti-HLA.A2 antibodies was performed as described previously [25]. Briefly, a LAB screen hybrid assay (One Lambda, a brand of Thermo Fisher Scientific, Canoga Park, CA, USA) and a LAB screen Single Antigen (One Lambda) on a LAB scan 3D system (One Lambda) were used according to the manufacturer’s specifications. Results are presented as median fluorescence intensity (MFI).

### 4.6. Flow Cytometry Analysis 

Freshly isolated spleen cells were obtained by careful grinding of mouse spleen in phosphate-buffered saline (PBS). Bone marrow cells were collected from the femurs of each mouse. Collected cells (1 × 10^6^ cells/mL) were stained with anti-B220-efluor 450 (clone RA3-6B2, eBioscience, San Diego, CA, USA), anti-IgM-APC (clone 11/41, eBioscience), anti-IgD-PE (clone 11-26C, eBioscience), anti-CD21/CD35-APC (clone 8D9, eBioscience), anti-CD38-FITC (clone 90, eBioscience), and anti-CD138-PE/Cyanine7 (clone 281-2, Biolegend) monoclonal antibodies to observe different B cell subsets (Table S2). For observation of T cell subsets, cells were stimulated with phorbol 12-myristate 13-acetate (Sigma) and ionomycin (Sigma) for 4 h, followed by the addition of GolgiStop (BD Bioscience, San Jose, CA, USA). Intracellular staining was performed using an intracellular staining kit (BD Biosciences or eBioscience) according to the manufacturer’s protocol. Stimulated cells were stained with the following antibodies: anti-CD4-FITC (clone RM4-5, eBioscience), anti-IFN-γ-APC (clone XMG1.2, eBioscience), anti-IL-4-PE-Cy 7 (clone BVD6-24G2, eBioscience), anti-IL-17-PE (clone 17B7, eBioscience), anti-Foxp3-APC (clone FJK-16S, eBioscience), and anti-CD25-eFluor 450 (clone PC61.5, eBioscience). Flow cytometric analysis was performed using a fluorescence-activated cell sorter (FACS) CantoII instrument (BD Biosciences). Data were analyzed using Flow Jo version 10.0.6 software (Tree Star, Ashland, OR, USA).

### 4.7. Quantitative Real-Time PCR (qRT-PCR)

Total RNAs were extracted from mouse spleen using an RNA isolation reagent (RNA-Bee;Tel Test, Inc., Friendswood, TX, USA). Five micrograms of purified RNA were transcribed into complementary first-strand DNA using a Dyne 1st-strand cDNA Synthesis Kit (Dyne Bio Inc., Seongnam, Republic of Korea). RT-qPCR amplification was performed using a SYBR Green Premix in a Light Cycler 480 system (Roche, Rotkreuz, Switzerland). Relative mRNA expression levels were normalized to the β-actin gene using the cycle threshold change method. Primer sequences used for qPCR are listed in Table S3.

### 4.8. Statistical Analysis

All data are expressed as mean ± standard error. Comparisons between groups were made by one-way ANOVA followed by Bonferroni post hoc tests. Differences with *p*-values less than 0.05 were considered significant. All statistical analyses were performed using GraphPad Prism version 5 (GraphPad Software, Inc., San Diego, CA, USA). 

## Data Availability

The data sets generated in this study are available from the corresponding author upon reasonable request.

## Supplementary Materials

The following supporting information can be downloaded at: https://www.mdpi.com/article/10.3390/ijms25031378/s1**.**
