# Supplementary material for: Combined Use of Tocilizumab and Mesenchymal Stem Cells Attenuate the Development of an Anti-HLA-A2.1 Antibody in a Highly Sensitized Mouse Model
Source: Int J Mol Sci. 2024 Jan 23;25(3):1378. doi: 10.3390/ijms25031378 (PMC10855827; doi:10.3390/ijms25031378)
Supplement: Supplementary file 1 [file ijms-25-01378-s001.zip › ijms-2789756-supplementary.pdf]

TABLES

Table S1. Definition of experimental group

Group	Name	Description
1	Syn-CONT	B/6→B6 mouse x 2 times
2	Allo-CONT	HLA→B/6 x 2 times
3	BM-MSC	Allo+ hBM-MSC 2x10^5/mice (every week)
4	TCZ	Allo+TCZ 10mg/kg (x3/week)
5	TCZ+BM-MSC	Allo+hBM-MSC 2x10^5/mice (every week)<br>+TCZ 10mg/kg (x3/week)

Abbreviation: HLA, Human Leukocyte Antigen;

Table S2. Fluorescence-activated cell sorting (FACS) markers

B cell subset	Surface marker
Pre-Pro B cell (BM)	B220+CD21/CD35-IgM-
Immature B cell (BM)	B220+CD21/CD35-IgM+
Mature B cell (BM)	B220+ CD21/CD35+IgM+
Long-lived Plasma cell (BM)	B220lowCD138+CD38low (Ig–)
Transitional B cell (SP)	B220+CD21/CD35loIgM+
Marginal B cell (SP)	B220+ CD21/CD35hiIgM+
Follicular B cell (SP)	B220+ CD21/CD35+IgM+
Memory B cell (SP)	B220+CD138-CD38+IgM-IgD-

T cell subset	Surface marker
Th1 (SP)	B220+CD21/CD35-IgM-
Th2 (SP)	B220+CD21/CD35-IgM+
Th17 (SP)	B220+ CD21/CD35+IgM+
Treg (SP)	B220lowCD138+CD38low (Ig–)

Table S3. Primer sequences used for qPCR

Genes	Primer sequence(5'-3')Forward	Primer sequence(5'-3')Reverse
IL-10	5'-CCAAGCCT-TATCGGAAATGA-3'	5'-TTTTCACAGGGGA-GAAATCG-3'
IL-23	5'-CAGCAGCTCTCTCGGAATCTC-3'	5'-TGGATACGGGGCACATTATTTTT-3'
IFN-y	5'-TCAAGTGGCATAGATGTGGAAGAA-3'	5'-TGGCTCTTGCAGGATTTTCATG-3'
Foxp3	5'- CAGCTGCCTACAGTGCCCCTAG-3'	5'-CATTTGCCAGCAGTGGGTAG-3'
